# Process evaluation of eHealth interventions for healthcare workers with psychological distress

**DOI:** 10.1192/j.eurpsy.2025.1428

**Published:** 2025-08-26

**Authors:** B. García-Vázquez, C. Bayón Pérez, M. F. Bravo Ortiz, R. Mediavilla Torres, J. M. Haro, M. Felez-Nobrega, J. L. Ayuso Mateos

**Affiliations:** 1 Psychiatry, Clinical Psychology and Mental Health, Hospital Universitario La Paz (HULP); 2 Psychiatry, Instituto de Investigación Sanitaria La Paz (IdiPAZ); 3 Psychiatry, Universidad Autónoma de Madrid (UAM); 4 Centro de Investigación Biomédica en Red de Salud Mental (CIBERSAM); 5 Instituto de Investigación Carlos III; 6 Hospital Universitario La Princesa; 7 Instituto de Investigación Sanitaria del Hospital Universitario La Princesa, Madrid; 8 Parc Sanitari Sant Joan de Deu (IRSJD); 9 Centro de Investigación Biomédica en Red de Salud Mental (CIBERSAM), Barcelona; 10 Psychiatry, Hospital Universitario La Princesa, Madrid, Spain

## Abstract

**Introduction:**

Over the past two decades, public health research has increasingly informed decision-making, but more evidence is needed on implementing interventions in real-life conditions. The COVID-19 pandemic highlighted these challenges, particularly in mental health. A study in Spain (RESPOND-HCWs) adapted and implemented psychological interventions for healthcare workers during the pandemic, showing significant reductions in anxiety and depression symptoms. This process evaluation aims to guide the practical implementation of these interventions in crisis settings.

**Objectives:**

This study provides a process evaluation of a successful stepped-care program involving eHealth interventions (Doing What Matters in Times of Stress [DWM] and Problem Management Plus [PM+]) designed for healthcare workers experiencing psychological distress (REsponding to Psychological distress among HCWs [RESPOND-HCWs] trial) conducted in Spain. The aim is to analyze program delivery context, assess implementation outcomes, and explore mechanisms of action.

**Methods:**

We utilized mixed methods, gathering quantitative data through routine RCT monitoring and structured observation, complemented by qualitative insights from semi-structured interviews with trial participants (n = 12) and decision-makers (n = 7), and a focus group discussion involving intervention providers (n = 7). Quantitative data were analyzed descriptively using R Software. Qualitative data underwent thematic analysis with NVivo.

**Results:**

Context analysis identified implementation barriers, such as DWM visual design, confidentiality, time to practice, stigma, unrealistic expectations about stepped-care programme, DWM phone calls, trust in the health system, internet literacy and intervention provider profile; and enabling factors, such as flexibility in schedules, remote delivery and mental health awareness initiatives. The implementation of the interventions was feasible, appropriate, and had minimal protocol deviations, with good participant acceptance. Mechanisms of action included confidence in intervention effects, a strong therapeutic relationship, and specific intervention components.

**Conclusions:**

The results serve as a complement to outcome evaluation and can guide wider implementation in similar settings. Recommendations include raising awareness about mental health and reducing stigma, providing brief orientation sessions, offering flexible schedules, and encouraging peer support.

**Disclosure of Interest:**

None Declared

